# Building a reference transcriptome for *Juniperus squamata* (Cupressaceae) based on single-molecule real-time sequencing

**DOI:** 10.1186/s12863-021-01013-x

**Published:** 2021-12-05

**Authors:** Yufei Wang, Siyu Xie, Jialiang Li, Jieshi Tang, Tsam Ju, Kangshan Mao

**Affiliations:** Key Laboratory of Bio-Resource and Eco-Environment of Ministry of Education, College of Life Sciences, State Key Laboratory of Hydraulics and Mountain River Engineering, Sichuan University, Chengdu, 610064 China

**Keywords:** *Juniperus squamata*, Single-molecule real-time sequencing, Simple sequence repeats, Gene ontology annotation

## Abstract

**Objectives:**

Cupressaceae is the second largest family of coniferous trees (Coniferopsida) with important economic and ecological values. However, like other conifers, the members of Cupressaceae have extremely large genome (> 8 gigabytes), which limited the researches of these taxa. A high-quality transcriptome is an important resource for gene discovery and annotation for non-model organisms.

**Data description:**

*Juniperus squamata*, a tetraploid species which is widely distributed in Asian mountains, represents the largest genus, *Juniperus*, in Cupressaceae. Single-molecule real-time sequencing was used to obtain full-length transcriptome of *Juniperus squamata*. The full-length transcriptome was corrected with Illumina RNA-seq data from the same individual. A total of 47,860 non-redundant full-length transcripts, N50 of which was 2839, were obtained. A total of 57,393 simple sequence repeats were identified and 268,854 open reading frames were predicted for *Juniperus squamata*. A BLAST alignment against non-redundant protein database was conducted and 10,818 sequences were annotated in Gene Ontology database. InterPro analysis shows that 30,403 sequences have been functionally characterized against its member database. This data presents the first comprehensive transcriptome characterization of *Juniperus* species, and provides an important reference for researches on the genomics and evolutionary history of Cupressaceae plants and conifers in the future.

## Objective

Compared with other plant groups, the genome analysis of coniferous species lags behind because of their larger genome [1, 2]. At present, only a few genome-wide datasets are available, such as *Sequoiadendron gigantea*, *Pinus taeda* L. and *Picea abies* [3–5]. Whole genome sequencing of conifers is prohibitively expensive for large genome sizes, and it also produces datasets which are inconvenient to analyze. In contrast, analyses on the dataset produced by transcriptome sequencing is much easier, and it is a convenient and cost-effective method for sequencing coding sequences of complex genomes.

*Juniperus squamata* is an evergreen shrub of the family Cupressaceae reaching 1–3 m tall, with brownish-gray bark [6]. It is found in mountains from southwestern China to northeastern Afghanistan, with separate populations east to Fujian and north to western Gansu in China [7]. This tetraploid species is not only of great value to gardening but also of enormous ecological values in subalpine and alpine shrubland ecosystems in Asian mountains. However, very limited genomic information is available for this species. Hence the objective of this work is to generate full-length transcriptome sequences for *Juniperus squamata*. Considering the importance of simple sequence repeats (SSRs) to plant population genetic analysis, we also developed SSRs for this species [8, 9]. To functionally characterize the full-length transcriptome, open reading frame (ORF) prediction and Gene Ontology (GO) annotation analysis were performed [10]. To functionally analyze the protein, the final isoforms were searched against InterPro’s predictive models [11]. The full-length transcriptome data set of *Juniperus squamata* can provide an important reference for its downstream analysis, such as genomic basis of environmental adaptation and genome evolution of Cupressaceae and even conifers.

## Data description

Fresh leaves, stems, and strobiles of one *Juniperus squamata* individual were collected from Kangding, Sichuan Province, China. For each tissue, the short paired reads were sequenced by Illumina platform. We also mixed the samples of each tissue and generated the long reads by the PacBio Sequel platform. Total RNA of the samples was isolated using the Plant RNA kit (Omega bio-Tech., USA) and then treated with RNase-free DNase I (NEB) to remove DNA. RNA degradation and contamination were monitored on 1% agarose gels and RNA purity was checked using the NanoPhotometer® spectrophotometer (IMPLEN, CA, USA). RNA concentration was measured using Qubit® RNA Assay Kit in Qubit® 2.0 Fluorometer (Life Technologies, CA, USA). RNA integrity was assessed using the Bioanalyzer 2100 system (Agilent Technologies, CA, USA). The Single-molecule real-time (SMRT) bell library was constructed with the Pacific Biosciences DNA Template Prep Kit 2.0 and SMRT sequencing was then performed on the Pacific Bioscience Sequel System. The sample used for Illumina sequencing was harvested using the same methods. The library was constructed using Illumina HiSeq X Ten. Adapter clipping and quality filtering of the Illumina raw reads was done using Trimmomatic version 0.36 [12]. Based on the quality check, the last two base pairs from each read were removed to minimize the overall sequencing error.

The raw full-length transcriptome sequencing data of samples were processed using the SMRT link version 4.0 software (https://www.pacb.com/support/softwaredownloads). Subread BAM files were generate from raw reads, parameters: -minLength 200, −minReadScore 0.75. Circular consensus sequence (CCS) was generated from subread BAM files, parameters: -min_length 50, −max_drop_fraction 0.8, −no_polish TRUE, −min_zscore − 9999.0, −min_passes 2, −min_predicted_accuracy 0.8, −max_length 15,000. CCS BAM files were output, which were then classified into Full-Length non-chimeric (FLNC) and non-full length (NFL) fasta files by examining the 5′ and 3′ adapters and the poly(A) tail. Iterative Clustering and Error Correction (ICE) algorithm was utilized to cluster FLNC fasta files to obtain cluster consensus. Quiver from SMRT link (parameters: -hq_uiver_min_accuracy 0.99, −bin_by_primer false, −bin_size_kb 1, −qv_trim_5p 100, −qv_trim_3p 30) were then utilized to polish cluster consensus sequence with NFL fasta files to obtain polished consensus sequence.

To obtain high quality corrected consensus sequence, additional nucleotide errors in polished consensus sequence were corrected using the Illumina RNA-seq data obtained from the same individual with the software LoRDEC version 0.7 [13] (parameters: -k 23 -s 3). Any redundancy in corrected consensus sequence was removed by CD-HIT version 4.6.1 [14] (parameters: -c 0.95 -T 6 -G 0 - aL 0.00 -aS 0.99 -AS 30) to obtain final a set of unique transcript isoforms. Benchmarking universal single-copy orthologs (BUSCO) version 3 was used to assess the quality of final transcript isoforms [15]. The summary statistics and length distributions of the PacBio SMART sequencing are shown in Data file 1 (Table S1 and Fig. S1). The results of BUSCO are shown in Data file 1 (Table S2). All three data sets obtained and their NCBI GenBank Accession numbers are listed in Table 1 (Data set 1, Data set 2, and Data set 3).

**Table 1:** Overview of data files/sets

Label	Name of data file/data set	File types (file extension)	Data repository and identifier (DOI or accession number)
*Data file 1*	*Summary and assessment of the data set*	*MS Word file(.docx)*	*Figshare (*10.6084/m9.figshare.14572125*)* [19]
*Data file 2*	*SSRs of Juniperus squamata*	*MS Excel file(.csv)*	*Figshare (*10.6084/m9.figshare.14572098*)* [20]
*Data file 3*	*Longest open reading frame prediction*	*gff3(.gff3)*	*Figshare (*10.6084/m9.figshare.16870147*)* [21]
*Data file 4*	*Alignment results of Juniperus squamata*	*text(.tab)*	*Figshare (*10.6084/m9.figshare.16870333*)* [22]
*Data file 5*	*Go annotation results of Juniperus squamata*	*text(.annotation)*	*Figshare (*10.6084/m9.figshare.16870401*)* [23]
*Data file 6*	*InterPro analysis results of Juniperus squamata*	*gff3(.gff3)*	*Figshare (*10.6084/m9.figshare.16912615*)* [24]
*Data set 1*	*js.fastq.gz*	*fastq (.fastq.gz)*	*NCBI(SRR13966305)* [25]
*Data set 2*	*juniperus_squamata_final.fastq.gz*	*fastq (.fastq.gz)*	*NCBI(SRR13993906)* [26]
*Data set 3*	*Juniperus_squamata_final_unique_transcript_isoforms.fastq.gz*	*fastq (.fastq.gz)*	*NCBI(SRR14000623)* [27]

MISA version 1.0 was employed to identify SSRs from final unique transcript isoforms of *Juniperus squamata* [16](parameters: definition (unit_size, min_repeats): 1–10 2–6 3–5 4–5 5–5 6–5, interruptions (max_difference_betw-een_2_SSRs): 100). Finally, 57, 393 SSRs were identified which were containing in 42, 273 sequences. The details of SSRs of *Juniperus squamata*, including primer sequences, SSR type, annealing temperature, product size etc., are shown in Data file 2. TransDecoder version 5.5.0 (https://github.com/TransDecoder/TransDecoder) was employed to identify ORF within the transcripts of *Juniperus squamata*. The results of ORF prediction are shown in Data file 3.

DIAMOND version 2.0.9.147 was used to align the final unique transcript isoforms against non-redundant protein database with a significance threshold of E ≤ 10^− 5^ [17]. A custom python (https://www.python.org/) script was used to carry out GO annotation (available at https://github.com/shanzha09/GO-annotation.git). InterProScan version 5.52–86.0 was used to search the final isoforms against interPro database [18]. The results of BLASTX alignment, GO annotation, and interPro analysis are shown in Data file 4, Data file 5, and Data file 6, respectively.

## Limitations

There is a shortcoming that we only collected one sample for single-molecule real-time sequencing of transcriptome.

## Data Availability

The data described in this Data note can be freely and openly accessed on *NCBI* under *SRR13966305, SRR13993906 and SRR14000623*. Please see Table 1 and references *Data file 1, 2, 3, 4, 5 & 6 and Data set 1, 2 & 3* for details and links to the data.

## Abbreviations

BUSCO: Benchmarking universal single-copy orthologs
CCS: Circular consensus sequence
FLNC: Full-length non-chimeric
ICE: Iterative Clustering for Error Correction
NFL: Non-full length
ROI: Reads of insert
SMRT: Single-molecule real-time
SSRs: Simple sequence repeats
